# National Association of Railway Surgeons

**Published:** 1889-06

**Authors:** 


### THE NATIONAL ASSOCIATION OF RAILWAY SURGEONS.

*Second Annual Meeting held in St. Louis, Missouri, May 2nd and 3rd.*

FIRST DAY—MORNING SESSION.

The Association was called to order by the President, Dr. J. W. Jackson, of Kansas City, at 10:30 A. M.

An address of welcome on behalf of the local profession was delivered by Dr. E. L. Gregory. He said the advance in modern surgery comes to wipe away the shame of multilation, once so humiliating in railroad injuries. The discovery of the causes of inflammation, and the methods of prevention have revolutionized medical science. Amputation, once the rule, is now the exception. Life in a limb is now a guarantee of its preservation.

DR. J. B. MURDOCH, of Pittsburgh, Pa., contributed a paper entitled

TORSION OF ARTERIES FOR THE ARREST OF HÆMORRHAGE, which appears in another part of this journal.

DR. S. D. ROBBINS, of Vicksburg, Mississippi, asked whether torsion could be resorted to in an artery so important as the femoral instead of ligation, with equally as good results.

DR. MURDOCH replied in the affirmative; he had applied torsion in the case of the femoral artery ninety-five times with success.

DR. GOODE, of Birmingham, Alabama, would not abandon the ligature entirely for torsion, but would rely on torsion in certain cases.

DR. P. FRENCH, of Mexico, Mississippi, had seen Bryant use torsion, and in order that the members might have a correct understanding as to the number of twists or turns that he makes, he would say that Bryant recommends making turns until the surgeon feels a want of resistance in the vessels.

DR. W. A. MCCANDLES, of St. Louis, held that the pedicle of tumors and the cat-gut ligature become living tissue.

DR. S. S. THORN, of Toledo, Ohio, challenged the statement made by D. McCandles. He believes that they were absorbed, and that we have evidence of this in operations where the ligature is used in closing surface wounds.

DR. G. J. NORTHROP, of Marquette, Michigan, that, in closing the discussion Dr. Murdoch state in what cases he would apply torsion, and in what cases he would select the ligature.

DR. R. HARVEY REED, of Mansfield, Ohio, said the question of what becomes of a ligature in ligating vessels had been pretty thoroughly demonstrated by experimentation. Dr. Senn, of Milwaukee, Wisconsin, had demonstrated that it was absorbed in experimenting on animals. The silk ligature, however, becomes encysted in from twenty to thirty days after the animal is killed. He had himself demonstrated this by experimentation. In the use of the cat-gut suture in abdominal surgery, unless the closure of the wound is rapid, the cat-gut becomes absorbed. He had never known the silk ligature to become absorbed and fail to do its duty.

With all due deference to Dr. Murdoch, he was a little afraid of torsion. He had experienced little or no difficulty with antiseptic silk sutures.

DR. NYE, of Pennsylvania, asked whether Dr. Murdoch had an opportunity of examining an artery, several days after he had applied torsion, to know its condition.

Dr. J. H. Murphy, of St. Paul, would hereafter try torsion, and after he did that he would apply a silk ligature as a means of further safety.

Dr. Murdoch, in closing the discussion, said it matters not whether torsion or ligation be resorted to, the principle is the same. With regard to the twisting of arteries, he finds that it requires four or five, or possibly more, revolutions of the forceps to make the necessary resistance. The surgeon knows that where a ligature is applied to an artery, whether it be silk, or cat-gut, it ruptures the internal and middle coats. It is the external coat that resists the ligature. There is retraction and contraction of the inner and middle coats, and a clot is formed, and it is the final organization of this clot that arrests the hæmorrhage.

His reasons for preferring torsion to ligature, either silk or cat-gut, are

1. On account of the greater facility with which it can be applied.

2. If the surgeon uses cat-gut the knot will untwist; it becomes softened before the clot is organized, and in that case secondary hæmorrhage is likely to occur.

3. With the experience of both in Guy's Hospital, London, and the Western Pennsylvania Hospital, it is safer.

First Day—Afternoon Session.

The Association re-assembled at 2 P. M., with President Jackson in the chair.

First-Vice-President Murphy was called to the chair, and President Jackson delivered his address, taking for his text

THE RAILWAY HOSPITAL SYSTEM OF THE MISSISSIPPI VALLEY.

The address was practically a history of the railway hospital movement, which, according to the speaker's data, originated in the West. The first consideration he had given to the subject of the establishment of a railroad hospital service was in 1867, during his incumbency of the office of local surgeon of the Missouri Pacific, at Kansas City. But there his surroundings forbade making such an attempt, although he did make the effort in 1872, as chief surgeon of the Atlantic and Pacific Railway Company in a territory extending from Pacific, Mo., to Vinita, I. T., and from St. Louis to Atchison, Kan. His first equipment was crude, and the success of the plan limited, owing to the lack of proper facilities for competent instant attention to persons injured on railroads, as well as for want of subsequent attendance and nursing. It was in the summer of 1879 that Dr. Jackson persuaded Superintendent Talmage, of the Wabash railroad, to make an attempt to establish a railroad hospital service on the assessment plan, which to-day has the almost universal indorsement of not only railway employés, but of the public at large.

The President's address was fully discussed: arguments were advanced for and against the establishment of such union hospitals.

A copy of the address was ordered to be sent to every railroad manager throughout the country.

The following resolution, offered by Dr. John E. Owens, of Chicago, was adopted without debate:

Whereas, During the past few years there have been organized in different parts of the United States several chartered corporations styling themselves, "The National Benefit Association," "The Mutual Aid Hospital Association," "The Sanitarum," and the like, offering, for small considerations, $6 to $10 annually, to issue certificates to members agreeing to furnish, in the event of sickness or accident, board, lodging, medicine, nursing, medical and surgical attendance; and

Whereas, Contracts have been made, and are being made, with the hospitals in towns and cities throughout the United States to furnish divers persons, holding the certificates of such associations, board, lodging, nursing, medical and surgical attendance, all necessary surgical instruments and medicines of the best quality, under such regulations as the said association shall from time to time prescribe, for the compensation of $1 per day in wards or $10 per week in private rooms; and

Whereas, We have already sufficient abuse of charity without permitting the development of another enterprise which thrives at the expense of hospital physicians and surgeons, who, per force of contracts made by said associations on the one hand and by the hospitals on the other, must turn out at all hours of the night, without compensation, to further the interests of the "one man" institution for whose pecuniary benefit the scheme is devised;

*Resolved*, That the members of the National Association of Railway Surgeons resent this new imposition upon the staffs of the various hospitals; that they acquaint the hospital authorities with the injustice of the scheme, and urge them to refuse such contracts as oblige their fellow-laborers in true charity to render professional services to the above-named benefit associations without compensation.

Dr. W. B. Outten, of St. Louis, read a paper entitled

A PLEA FOR MORE DETAIL IN THE TREATMENT OF RAILWAY INJURIES.

Dr. T. E. Russell, of Springfield, Ohio, read a paper on

RAILWAY SURGERY, in which he advocated primary amputation. He said in severe injuries to the arm near the shoulder, requiring amputation, it often happens that the lesion is so near the articulation that it is next to impossible to secure a good flap by any methods laid down in text-books. A convenient and proper way, if the sound tissues favored the scheme, is to make the first incision in the course of the humerus, splitting the deltoid muscle and going directly to the shoulder joint. A strong bistoury is introduced below and a little in front of the acromion process and a longitudinal cut is made in the course of the limb to a point an inch and a half below the surgical neck of the humerus. At the middle of this incision the scalpel is placed in the wound, and carried obliquely downward in front and behind, making two lateral flaps, as it were. These lateral cuts should not be deep enough in front or towards the axilla to sever the arteries, but to make space for disarticulating the head of the humerus from the glenoid cavity. The remainder of the amputation is completed by returning to the lateral or oblique incisions—to the posterior first—and cutting each away until the arm is severed. The flaps can easily be adjusted, and the result is usually satisfactory, if the healing processes meet with no mishaps.

Photographs of the cases on which Dr. Russell had thus operated, were exhibited, showing the results.

Dr. H. H. Middlecamp, of Warrenton, Missouri, advocated primary amputation where there was no possible chance of saving the limb. Injuries produced by railway cars were more profound and severe than those received otherwise, owing to the relative speed, the weight of the cars, etc. There is very little difference, however, between some railway injuries and those sustained in large manufacturing establishments where there is powerful machinery. The surgeon should exercise his judgment as to whether amputation should be performed immediately or delayed.

Dr. A. W. Atchison, of Denison, Texas, said it was surprising to find what little support the surgeon had in regard to primary amputation. He had searched the different authorities on this subject. Personally, he would favor immediate operation.

Dr. R. Harvey Reed, of Mansfield, Ohio, was in favor of primary operation. When the surgeon waits for shock in severe railway injuries, or in a case where heavy machinery has gone over a limb, he only waits for the undertaker, for almost invariably the patient dies.

Dr. J. H. Bennett, of Wauseon, Ohio, advocated primary amputation. One of the principal elements of reaction and repair is rest. The surgeon should, therefore, put the patient at rest as soon as possible after the injury. This could not be done, however, in the case of a badly mutilated limb, and the surgeon should perform amputation before reaction sets in.

Dr. J. H. Murphy, of St. Paul, had been a railroad surgeon for twenty-four years. The time to operate depended wholly and solely on the judgment of the surgeon. A man without judgment was not fit to be a railroad surgeon. He did not think there was any surgeon who could go into court and testify when an amputation should be done.

Dr. W. L. Schenck, of Osage City, Kansas, protested against one thought brought out in the paper: *that the operation should be performed immediately.* It was misleading to the young surgeon.

Dr. von Mansfeld, of Nebraska, called attention to the influence upon the nervous system and brain as a distinction between railway and military surgery. The surgeon has no more hopeful subject to deal with than the boy who works on a railroad; whereas, in military surgery the excitement during engagements is such that any injury is accompanied by a severe shock that may kill the patient. Where a patient has sufficient vitality to withstand an operation, he would not hesitate to perform it.

Dr. Allen, of Liberty, Missouri, said gunshot wounds were rarely followed by much hæmorrhage. He had never seen a severe hæmorrhage during the whole war; whereas, in railway injuries one of the prominent features is hæmorrhage. This, he thought, was a striking distinction between railway and military surgery.

With regard to the propriety of operating, he presumed no surgeon would operate on a pulseless patient, or one in whom collapse would follow shock. When, however, the indications were such as to justify an operation, he would certainly do it.

*Shock*, he thought, could be overcome by alcoholic stimulants, coupled with large doses of morphine, one-half of a grain of the latter, if necessary, followed by the use of chloroform. He believed that ninety-five per cent. of all the cases of shock could be thus overcome. If the pulse rallies under chloroform he regards it as a favorable sign.

Dr. S. S. Thorn, of Toledo, Ohio, would operate if the patient rallied under an anæsthetic. He then referred to the septic period. Somebody wrote "*Woe to the surgeon who operates in this period.*" According to his observations, patients operated upon during the septic period, as a rule, died.

Dr. Outten said there was one element that had borne him down and which he had been thoroughly incompetent to treat, and that was *shock*. He would venture to say in violent railway injuries that thirty-three per cent. of the cases die of shock before the surgeon can interfere.

Dr. C. B. Miller, of Lawrenceburg, Indiana, said in railway injuries and accidents the shock is greater, for the reason that in a large number of cases those who are injured on railroads see the object that is coming; while those who are wounded in the army are wounded without knowing they have been struck until a short time after.

He could not agree with Dr. Allen who had said that gunshot wounds rarely gave rise to much hæmorrhage. He had made a great mistake, for they were much more liable to bleed than some of the injuries received on railroads.

First Day—Evening Session.

Dr. Reed read a communication from the Secretary of the American Public Health Association, requesting that some member be appointed to prepare a paper on Railway Sanitation, to be read at the next annual meeting of the Association, to be held in Brooklyn, New York, October 23–24–25.

No action was taken on the communication.

Dr. Robert Barclay, of St. Louis, read a paper entitled

THE WHISTLE SIGNAL, in which he recommended aural tests to be scientifically applied to all railroad employés, just as color tests are now applied.

Dr. Murdoch thought the suggestions offered were good. The engineer should have a good ear as well as a good eye, and considerable attention had been given to the latter of late in the employment of engineers, firemen, and pilots on vessels. The subject of the ear should in future engage the attention of railway surgeons, and he would suggest that they be included under one head, if it is thought well to act on Dr. Barclay's suggestions, as ophthalmology and otology usually go together.

Dr. Adolph Alt, of St. Louis, read a paper entitled

PTERYGIUM AMONG RAILROAD MEN, which was referred to the Committee on Publication.

Recording Secretary Stemen made an appeal for the spread of the sentiments which gave rise to the formation of the Association.

The Corresponding Secretary read a few of the many letters he had received from the general managers of railroads pertaining to the organization, the tenor of which was very favorable to the Association.

May 3rd, Second Day—Morning Session.

Dr. A. W. Atchison, of Denison, Texas, read a paper on

RAILROAD STRETCHERS.

The paper was discussed by Drs. Mansfeld, Outten, and A. W. Ridenour of Massillon, Ohio.

A further discussion on the establishment of union hospitals followed at this juncture, which was participated in by President Jackson, Dr. W. P. King of Kansas City, and Dr. Ridenour.

Dr. Fulton, of St. Paul, read a paper on Railroad Injuries of the Eye.

Second Day—Afternoon Session.

Dr. Reed contributed a paper entitled

CLINICAL OBSERVATIONS ON SOME OF THE EFFECTS OF DIRECT AND INDIRECT TRAUMATISMS OF THE BRAIN.

The paper was not discussed.

Officers for 1890.

*President*—Dr. J. B. Murdoch, Pittsburgh, Pennsylvania.

*First Vice-President*—Dr. W. B. Outten, St. Louis, Missouri.

*Second Vice-President*—Dr. Elliott, Atlanta, Georgia.

*Third Vice-President*—Dr. W. B. Rogers, Memphis, Tennessee.

*Fourth Vice-President*—Dr. J. F. Pritchard, Manitowoc, Wisconsin.

*Fifth Vice-President*—Dr. G. P. Conn, Corcord, New Hampshire.

*Sixth Vice-President*—Dr. T. P. Livingston, Plattsmouth, Nebraska.

*Seventh Vice-President*—Dr. F. J. Bancroft, Denver, Colorado.

*Recording Secretary*—Dr. C. B. Stemen, Fort Wayne, Indiana.

*Assistant Secretary*—A. G. Gumaer, Buffalo, New York.

*Corresponding Secretary*—Dr. E. R. Lewis, Kansas City, Missouri.

*Treasurer*—Dr. R. Harvey Reed, Mansfield, Ohio.

The Association then adjourned to meet in Kansas City, Missouri, on the first Thursday in May, 1890.

